# Weight Stigma Goes Viral on the Internet: Systematic Assessment of YouTube Comments Attacking Overweight Men and Women

**DOI:** 10.2196/ijmr.9182

**Published:** 2018-03-20

**Authors:** Yongwoog Andrew Jeon, Brent Hale, Eric Knackmuhs, Michael Mackert

**Affiliations:** ^1^ Center for Health Communication Moody College of Communication The University of Texas at Austin Austin, TX United States; ^2^ The Media School Indiana University Bloomington Bloomington, IN United States; ^3^ School of Kinesiology, Recreation & Sport Western Kentucky University Bowling Green, KY United States

**Keywords:** stigma, cyberbullying, gender, sex differences, verbal behavior

## Abstract

**Background:**

Anonymous verbal attacks against overweight individuals on social media are common and widespread. These comments often use negative, misogynist, or derogatory words, which stigmatize the targeted individuals with obesity. These verbal attacks may cause depression in overweight individuals, which could subsequently promote unhealthy eating behavior (ie, binge eating) and further weight gain. To develop an intervention policy and strategies that tackle the anonymous, Web-based verbal attacks, a thorough understanding of the comments is necessary.

**Objective:**

This study aimed to examine how anonymous users verbally attack or defend overweight individuals in terms of 3 themes: (1) topic of verbal attack (ie, what aspects of overweight individuals are verbally attacked), (2) gender of commenters and targeted overweight individuals, and (3) intensity of derogation depending on the targeted gender (ie, the number of swear words used within comments).

**Methods:**

This study analyzed the content of *YouTube* comments that discuss overweight individuals or groups from 2 viral videos, titled “Fat Girl Tinder Date” and “Fat Guy Tinder Date.” The twin videos provide an avenue through which to analyze discussions of obesity as they organically occurred in a contemporary setting. We randomly sampled and analyzed 320 comments based on a coding instrument developed for this study.

**Results:**

First, there were twice as many comments verbally attacking overweight individuals (n=174) than comments defending them (n=89). Second, overweight women are attacked for their capacities (eg, laziness, maturity; 14/51, 28%), whereas overweight men are attacked for their heterosocial skills (eg, rudeness, annoyance; 24/29, 83%). Third, the majority of commenters who attacked overweight women are male (42/52, 81%). Fourth, attacking comments generated toward overweight women included more swear words (mean 0.44, SD 0.77) than those targeting men (mean 0.23, SD 0.48).

**Conclusions:**

Our data elucidate a worrying situation of frequent disinhibited aggressive messages against overweight individuals online. Importantly, the patterns of verbal aggression differ depending on the gender of the targeted overweight individuals. Thus, gender-tailored intervention strategies that specifically tackle Internet users’ verbal aggression against overweight individuals need to be developed.

## Introduction

### Background

Weight stigma—negative attitudes and beliefs toward individuals with obesity—is pervasive across societies [1]. Weight stigma often manifests stereotypes, rejection, and prejudice toward individuals with obesity. One of the most common forms of expressing weight stigma is through verbal attack or verbal bullying defined as statements that attack the self-concept of the receiver, intending to deliver psychological pain (eg, teasing, ridiculing, derogating, devaluing, humiliating). Research shows that verbal attacks on overweight individuals are common on social media. A recent study, which analyzed 1.37 million posts collected from various social media channels including YouTube, found that 92 percent of the posts related obesity with negative, misogynist or derogatory words [2]. Additionally, researchers suggest that weight-based aggressive comments are highly likely to induce depression or anxiety in overweight individuals [3]. Depression could subsequently result in binge eating and weight gain, which may increase exposure to further weight-stigma and, at worst, even lead to suicide [4]. Due to these adverse effects of weight stigma, various authors called for more intervention efforts, including development of health messages, to reduce weight stigma and relieve mental burden for individuals with obesity [5,6]. Effectiveness of health messages to reduce weight stigma largely depends on precise knowledge of the audience—those being stigmatized (ie, obese individuals) and those who stigmatize (ie, individuals who verbally attack individuals with obesity) [7,8]. Particularly, men and women may experience weight stigma differently [9]. Taking this into account, gender-tailored messages need to be developed since they enhance persuasion by increasing perceived relevance [10]. To develop health messages tailored to female and male audiences, it is important to understand how overweight women and men are stigmatized *differently* on social media, and how female and male commenters stigmatize overweight individuals differently.

This study asks: Which negative trait is most dominantly associated with overweight males or females? As evolutionary psychological scholars suggest, verbal aggression against men generally addresses men’s physical or mental strength (eg, laziness, physical weakness) while those against women tends to be about their appearance [11]. This study attempted to establish this topical difference in verbal attack as it applies to overweight individuals. Second, in terms of those who stigmatize, we attempted to find out what the major concerns of male and female *YouTube* users are when commenting on overweight individuals. Two similar videos (female and male Tinder videos), which were sequentially published to YouTube, provided an opportunity to minimize confounds due to the gender of the obese model in the video (ie, the female model may attract more comments targeting female overweight individuals or vice versa). Furthermore, these comments provide an opportunity to conduct a natural experiment to gain insight into how the commenters—which would not be made in a laboratory setting due to the social desirability issue—attack or defend individuals with obesity.

It is important to develop effective, gender-tailored intervention strategies that are fully informed by theoretical understanding and empirical findings on the verbal aggression targeting overweight individuals on the Internet. Thus, for this purpose, the study’s coding-instrument, based on linguistic and evolutionary psychological theories, was developed to analyze comments posted in response to the *YouTube* videos. In the following section, the theoretical framework for the analysis of the aggressive comments is discussed. On the basis of this review, the specific research questions are proposed.

### Gender Differences in Weight Stigma

Some cultures value obese human bodies as expressing beauty, marriageability, control of selfish desires, generosity, fertility, and closeness to God [1]. Yet across many contemporary cultures, obesity is strongly linked with various negative traits [1], which include laziness, unattractiveness, lack of will power, poor self-control, slowness, inactiveness, physical weakness, overeating, ineptitude, sexlessness, and unhealthiness [12,13]. Obese individuals have reported frequently experiencing verbal attacks that target these negative traits in their everyday lives [14] from strangers (eg, an overweight woman was called a “big fat pig” and physically assaulted by a stranger on a commuting train) [15] or from a spouse (eg, a husband called his wife “disgustingly fat”) [16]. More evidence has been recently accumulated to suggest that the association between negative traits and obesity is becoming a global issue [1].

However, most research on weight stigma has not explicitly addressed possible gender variations in associations of negative traits with obesity. By understanding how male and female overweight individuals are attacked, one may inductively infer how people’s attitude toward overweight males differs from that toward overweight females. For instance, in the context of gender differences, the following questions can be asked: Are overweight females attacked for laziness more frequently than obese males? Are obese males attacked for physical weakness more commonly than females? With a lack of current empirical research exploring these questions, this study draws upon evolutionary psychological views to provide theoretical grounding.

Evolutionary perspectives offer the basis for explaining (at a fundamental level) and predicting sex differences in verbal aggression. Scholars in evolutionary psychology claim that to maximize reproductive success (ie, the number of their offspring), males, on the one hand, evolved to infer a mate’s reproductive success from physical appearance, more so than females [11,17-20]. Healthy skin, hair, and low hip-waist ratio are some of the visual cues for *reproductive power* [17] *.* On the other hand, females evolved to infer a mate’s *capacities for resource acquisition* or the ability to provide protection or food [17]. Accordingly, females primarily sought mates with resource acquisition-signaling features most of which were not physically visible: including possession of territory, shelter, industriousness, or high social status [21]. These evolutionary psychological views have been supported by various studies examining contemporary people’s feature preferences in mates [22-24]. For instance, in a survey that sampled more than 1000 undergraduates, women responded that they value males’ education and financial prospect (ie, capacities for resource acquisition) more than “good looks” (ie, reproductive power), whereas men said that they value females’ physical attractiveness more than financial prospect [22].

Such gender difference in preferred features of mates may predict which personal characteristics will be targeted by aspects of verbal aggression. Buss and Dedden (1990) conducted an experimental study where they investigated what a man or woman would derogate about a perceived competitor for a mate. They found that when women verbally attacked other competing women, their reproductive power tended to be addressed in the verbal aggression (eg, “she is physically unattractive”) [11]. When men verbally attacked other competing men, their capacities for resource acquisition were likely to be addressed (eg, “the man lacked ambition” or “the other man is poor”). Informed by the evolutionary psychological perspective, the reproductive power and capacities for resource acquisition dichotomy is employed in this study. Utilizing this dichotomy, we examined verbally aggressive comments in relation to gender difference within the Tinder date videos. Videos and comments on *YouTube* addressing overweight individuals may show how they are stigmatized on social media [25,26]. While findings from previous studies are useful for understanding what generates stigmatic content on *YouTube*, various important questions regarding gender difference in weight stigma remain unanswered. Thus, the following research questions (RQ) were developed for this purpose:

RQ1a: To what extent are overweight women targeted regarding reproductive power more than men in aggressive comments within YouTube comments?

RQ1b: To what extent are overweight men targeted regarding capacities for resource acquisition more than women in aggressive comments within YouTube comments?

Other research suggests that people judge their romantic partners based on heterosocial skills [22], which generally refer to a person’s ability to carry a good conversation or express a good personality. Specifically, studies suggest that males are likely to succeed in finding a female mate if he is heterosocially skilled [27,28]. However, to our knowledge, little is known about how obese individuals’ heterosocial skills are perceived. Like other negative traits attributed to overweight individuals, heterosocial skills could be underestimated in order to maintain negative valence. To explore this possibility within weight-based verbal attacks in *YouTube* comments, the following research question is proposed:

RQ2: What gender differences exist in how overweight individuals’ heterosocial skills are attacked within YouTube comments?

Finally, among the 3 explored topics of verbal attack (ie, resource acquisition, reproductive power, and heterosocial skills), we explore gender-based differences in topical prominence:

RQ3: What gender differences exist between the prominence of attacks on reproductive power, capacities for resource acquisition, and heterosocial skill?

### Gender Difference in Susceptibility to Weight Stigma

Many researchers have suggested that obese women are more susceptible to weight stigma than men [9,29]. In a study conducted by Chen and Brown (2005), 449 college students were asked to order 6 pictures of potential sexual partners according to their personal preference [30]. The pictures included an obese person, a nonobese person, and individuals with various disabilities. A rating comparison between subjects showed that men rated an obese partner less preferentially than women did. In addition, men are more likely to choose sexual partners based on a potential partner’s weight. Overall, their findings showed that women may be more vulnerable to weight stigma exposure than men. In contrast to these findings; however, Hussin et al (2011) found that across the 50 most viewed *YouTube* videos which devalued overweight individuals, overweight men are twice as likely to be targets than overweight women [25].

Men are primarily considered the perpetrators of weight stigma in a masculine society, as for women “becoming an attractive object is a role obligation” [31]. Thus, men are less likely to feel guilty when they fulfill masculinity by victimizing women whom they perceive as deviant [32]. However, in the context of romantic relationships, Buss and Dedden (1990) argued that women evolved to engage in verbal aggression against their own sex since members of a sex must compete for access to a mate with desired qualities (eg, reproductive power or physical strength) [11]. A question may be raised as to whether this is the case for overweight individuals of the same sex or not. Unlike slender women, overweight women may be perceived as less physically attractive (and less of a competitor), and thus would not require verbal aggression as frequently. Alternatively, it is possible that women will defend overweight women due to contemporary cultural or social factors. Therefore, we sought to answer the following research questions regarding the presence of attack and defense between genders:

RQ4: *Which gender more frequently verbally attacks or defends overweight individuals? (a) men or (b) women?*

### Intensity of Derogation—Toxic Disinhibition Effects and Swearing

Many scholars claim that the Internet is an arena where people feel disinhibited to state what they would not ordinarily say face-to-face [33-36]. Suler (2004) argues that this is an affordance of anonymity online—more so than face-to-face communication— allowing Internet users to feel less restrained by the consequences of saying what is ordinarily socially undesirable, which includes swearing or expressing hatred against overweight people. This phenomenon is termed as “Toxic Disinhibition Effects” [36]. According to the perspective of Toxic Disinhibition Effects, people would feel less inhibited in expressing verbal aggression against individuals with obesity, potentially resulting in a plethora of derogatory or aggressive comments targeting obese people [2].

A body of evidence has supported the disinhibition effect [2,37,38]. For example, in a content analysis of 9376 Myspace pages by Thelwall (2008), it was found that the pages of nearly all young and half of middle-aged Myspace users contained swear words. Furthermore, Chou et al (2014) analyzed 2.2 million posts across Twitter and Facebook, and they found that keywords including “obesity,” “overweight,” and “fat” are associated with derogatory terms including “a**” or “b****.” Also, qualitative analysis of these posts revealed that the most prevalent theme is weight stigma (eg, “you’re an ugly fat b****. Kill yourself”) [2]. Within *YouTube*, these verbal aggressions were found to be common as well [38,39]. On the basis of this literature, the following research questions are formulated:

RQ5a: To what comparative extent do overweight individuals receive more verbal attacks than defense?

RQ5b: To what extent are verbal attacks accompanied by swear words?

RQ5c: To what comparative extent do male and female overweight individuals receive verbal attacks which include swear words?

## Methods

### Study Design

This study entailed a content analysis of *YouTube* comments that discuss overweight individuals or groups from 2 videos, titled “Fat Girl Tinder Date” and “Fat Guy Tinder Date.” These 2 videos were released approximately 3 weeks before the beginning of this study, providing an avenue through which to analyze discussions of obesity as they organically occurred in a digital setting. Utilizing this window of opportunity, the authors sought to evaluate how attacks toward the stigmatized may occur in this context.

### Background Information and Rationales for Choosing the Two Videos

On September 24, 2014, a video titled “Fat Girl Tinder Date” (hereafter the female Tinder video) [40] was published by a *YouTube* content creator. In this video, a slender woman set up a number of dates through Tinder, a matchmaking mobile app, using a profile picture which accurately depicted her appearance; however, before appearing for each date she disguised herself as an obese woman by wearing body adhesives. Hidden cameras videotaped how men reacted to the “obese” woman. Later, a similar video was released titled “Fat Guy Tinder Date” (hereafter the male Tinder video) [41], with a slender man attending Tinder-arranged dates wearing comparable body adhesives. After their release, these videos went viral, accumulating over 12 million views and approximately 4000 user-submitted comments within 3 weeks. One of the most popular comments (indicated by the number of “thumbs up” received) states “I f****** hate fat b****,” and another one says “If you’re fat just move your f****** a** and go to a gym”. The majority of obesity-related *YouTube* videos explicitly use weight-based teasing (eg, verbally mocking individuals with obesity or blaming them for their weight statuses) [26]. These videos, therefore, may promote negative comments on individuals with obesity [25].

The 2 videos chosen for this study exemplify the obesity-related *YouTube* videos that contain such a negative theme, though less explicitly [26]. Also, in general, the narrative structure of the 2 videos are similar; yet everything is not equal (eg, contents of conversations). Despite the potential confounds coming from difference between the contents of the 2 videos, these videos best serve our research purpose: the examination of gender difference in comments on obese individuals. This is in that the gender of the person with obesity may result in more comments about the traits that are stereotypically linked with obese individuals of the same gender (eg, a male obese model in the video promotes discussion of traits typically linked with an obese man than woman). As in our data, the Male Tinder video attracts significantly more male comments than the Female Tinder video and *vice versa* (Table 1) *.* Thus, to minimize the confounds coming from the gender of the model in the video, we chose the 2 “twin” videos to sample comments from.

More importantly, the comments that may explicitly verbally attack obese individuals may not be obtained in a survey or a laboratory experiment due to social desirability [42]. Instead, the 2 Tinder videos provided a setting for “a natural experiment” [43]. A natural experiment has been increasingly employed for research in social media contexts [16,44]. The producer purposefully made these 2 videos highly similar to see how different genders would react to the obese blind-date partners. Then they posted these 2 videos on *YouTube*, which inadvertently provided a setting for a natural experiment.

### Data Collection and Sampling

Approximately 3 weeks after the 2 videos were released, all available comments were captured and saved in 2 word processing files (1 for each video). The captured material included the commenter’s profile photo, the commenter’s username, the comment text, and the timestamp for the comment’s creation. Upon completion of data collection, the authors processed a random sample of 320 comments from the data. The random sample was procedurally selected through the utilization of a random number generator, which selected pages of the word processing file to analyze for relevant comments. All comments were then subjected to our inclusion criteria (explained in the next section), and rejected comments were replaced by continuing the selection process until a sample of 320 comments was obtained. Once the sample was collected, the authors conjointly reviewed the comments to achieve agreement that each entry passed our inclusion criteria, which left us with 316 verified comments to analyze. A total of 145 comments were selected from the “Fat Guy Tinder Date” video, while 171 comments were selected from the “Fat Girl Tinder Date” video.

### Inclusion Criteria

Each comment was subjected to a filter designed to allow analysis of only the relevant comments. The first criterion was that each comment needed to contain some reference to an individual or group being overweight or make a comment about overweight people generally. The second criterion was that each selected comment needed to make some statement about weight, including an opinion, criticism, or evaluation about being overweight or the experiences of being overweight. In addition, we intended to exclude those comments directed at the actor or actress, not overweight individuals in general. No comment in our dataset was found and excluded for this reason.

### Variables of Interest

Each of the following variables was developed for the purpose of this study, and intercoder reliability for each variable is reported based on Krippendorff alpha, a statistical measure of the interreliability among the coders for our variables [45]. The overall Krippendorff alpha is .85.

#### Gender of Commenter (Alpha=.70)

This study used the commenters’ names and profile pictures to infer gender. Although it could be posed that identification of true commenter gender cannot be accurately conducted within digital contexts due to potentially unidentifiable gender swapping, some research has shown that females and males tend to represent themselves accurately in their profile photo or name rather than engaging in gender swapping [46-48]. There may be other options (eg, directly contacting the commenters by leaving a message in their channel or “vlog” on *YouTube*). However, we realized that contacting more than 300 commenters was extremely difficult and time-consuming. Even if we contacted the commenters, perhaps due to potential social desirability issue, *YouTube* commenters would probably hesitate to provide their true identity (eg, gender). Thus, inference of gender from their uses of profile name and photos was the second best approach.

Commenter gender was coded when identifiable in the profile photo, username, or comment text. In many cases, gender was determinable in at least one of these locations, and no instances occurred where gender determination conflicted between locations (ie, male profile photo and female username occurring simultaneously). In terms of the profile photo, the 3 coders judged whether the face is male or female. In addition, we used the database on the names trending in America which is provided for researchers by US Social Security Administration [49]. This database matches names with gender (eg, Jake for male). When identifiable, commenters were coded as male or female; otherwise, indeterminable. Thus, we examined both usernames and profile photos to infer the gender of the commenter and compared this result with information within the comment text. To focus on our primary research purpose (examination of gender differences in the comments on overweight individuals), we excluded those comments whose profiles do not provide clear clues of gender identifications.

#### Gender of Target (Alpha=.88)

Comments were coded according to the gender of the target of the comment text. The target of the comment text was determined as the individual or group whose weight is being discussed or evaluated. In some instances many targets existed, while most included only a single target. When identifiable, this was coded as male, female, or both in cases where both genders were targeted by the comment; otherwise, neither.

#### Attack (Alpha=.93)

Comments were categorized as containing an attack when the comment text offended, belittled, or expressed disapproval of an individual or group being overweight. In cases where only portions of comment text concerned weight, only those sections that passed our inclusion criteria were evaluated for attack potential.

#### Defense (Alpha=.88)

Comments were categorized as containing a defense when the comment text protected or supported an individual or group’s weight, or an overweight individual’s actions, thoughts, or traits related to weight. As with attack, only those portions of comment text that passed the inclusion criteria were evaluated.

#### Evolutionary Mate Values (Alpha=.77)

This variable was operationalized in 2 distinct ways, resulting in 2 categories. A comment was categorized as an attack or defense of capacities for resource acquisition in cases where the following were targeted: (1) an overweight individual or group’s physical or mental ability and (2) personal characteristics of responsibility, laziness, health, or maturity. Comments were categorized as an attack or defense of reproductive power in cases where an overweight individual or group’s attractiveness, ability to find a mate, give birth, or raise a child are targeted. These categories were adapted from Buss and Dedden (1995).

#### Heterosocial Skills (Alpha=.71)

A comment was categorized as an attack or defense of relational skills when the comment text addressed an individual or group’s ability to interact with others, which included attacks on social skills or behavior, ability to be selected as a mate, and desirability.

#### Intensity of Derogation (Alpha=.89)

The number of swear words were counted and recorded to indicate derogation intensity.

## Results

### Overview

Within the selected data from the female Tinder video (n=171), 75 (44%) commenters were male, 37 (22%) were female, and 59 (35%) were indeterminable. For the male Tinder data (n=145), 45 (31%) commenters were male, 52 (36%) were female, and 48 (33%) interminable. The descriptive figures of the 2 Tinder videos are reported in Table 1.

**Table 1:** Overview of descriptive results (those interminable are excluded in the analysis and this table).

Gender of the actor in the video	Purpose		Target gender		Commenter gender	
	Attack	Defense	Male	Female	Male	Female
Male video, n (%)	71 (49)	43 (30)	73 (50)	33 (23)	45 (31)	52 (36)
Female video, n (%)	103 (60)	46 (27)	7 (4)	98 (57)	75 (44)	37 (22)
Total, n	174	89	80	131	120	89

In terms of analysis, all the research questions focus on the difference in frequency of occurrence (eg, number of comments that verbally attack). Thus, to analyze this dataset, chi-square analyses were employed, with the exception of intensity of derogation, for which we employed a *t* test that examined the mean differences.

### Evolutionary Mate Values—RQ1a and RQ1b

Our first research question asks up to what extent overweight women are verbally attacked according to reproductive power more than men. In addition, we ask up to what extent overweight men are attacked according to their capacities for resource acquisition more than women. We found no significant differences in the frequency of comments attacking physical appearance and resource acquisition between comments targeting male and female overweight individuals, χ^2^_1_(N=66)=2.8, *P*=.09. The majority of comments targeting overweight males attacked their reproductive power (14/19, 74%), while females were targeted for both resource acquisition (24/47, 51%) and reproductive power (23/47, 49%). These results indicate that when addressing evolutionary mate values, it is primarily reproductive power (ie, physical attractiveness) for which both genders are verbally attacked.

### Heterosocial Skills—RQ2

An exploration of our second research question requires an examination of the comparative extent up to which overweight males and females receive verbal attacks addressing heterosocial skills. A significant difference was found between male and female overweight targets, χ^2^_1_(N=138)=9.9, *P*=.002. Our results indicate that male targets are more likely to receive comments that attack their heterosocial or interpersonal skills. Of male targets, 87% (40/46) received comments about relationship deficits. Of female targets, 61% (56/92) received comments of this type.

### Topic of Verbal Attack Comparison—RQ3

To explore our third research question, we conducted a frequency comparison between attacks on capacities for resource acquisition, reproductive power, and heterosocial skills between male and female individuals. Initially, evolutionary mate values (ie, personal failings, which include resource acquisition and reproductive power) and heterosocial skills were coded separately, and therefore overlap between these 2 variables was allowed. That is, both evolutionary mate values and heterosocial skills could be expressed in a single comment. We combined evolutionary mate values and heterosocial skills into a single variable, Topic of Comment, to determine whether gender differences exist between the prominence of attacks on reproductive power, capacities for resource acquisition, and heterosocial skills.

Our results show that there exists a significant difference in the topic of aggressive comments between target gender, χ^2^_2_(N=80)=7.6, *P*=.02. On the basis of the standardized residuals of each comparison, we found that capacities for resource acquisition (residual=−4.8) and heterosocial skills (residual=4.6) are the two sources of significance, that is, female overweight targets are more likely to receive comments that attack capacities for resource acquisition (14/51, 28%) than male overweight targets (1/29, 3%). However, male overweight targets are more likely to receive comments that attack their heterosocial skills (24/29, 83%) than female overweight targets (29/51, 57%). Reproductive power in this case was not a source of difference between male and female targets (residual=−.2). When defending, there was no significant difference in the topic of comments between male and female overweight targets, χ^2^_2_(N=49)=1.1, *P*=.56.

### Target Gender—RQ4a and RQ4b

We asked which gender receives more verbal attacks within the 2 videos. No significant difference in the number of verbal attacks was found between the gender of targets, χ^2^_1_(N=211)=3.6, *P*=.06. To answer RQ4a and RQ4b, we explored which commenter gender tends to attack male or female overweight individuals. We found a significant difference between male and female commenters in attacking female overweight targets, χ^2^_1_(N=87)=5.7, *P*=.02 Male commenters are more likely to attack female overweight targets (42/52, 81%) than female commenters (20/35, 57%). Yet, when male overweight individuals are targeted, no difference in frequency of verbal attack was found between the commenter genders, χ^2^_1_(N=50)=1.2, *P*=.28. No significant difference between the commenter gender in the frequency of defense was found.

### Intensity of Derogation—RQ5a, RQ5b and RQ5c

**Figure 1:**
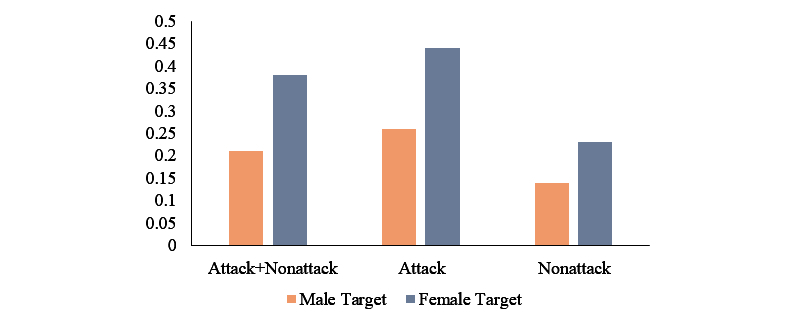
Average number of swear words (intensity of derogation). The y-axis indicates the number of swear words used in the comments. "Attack+Nonattack" refers to comments regardless of its valence. Attack and nonattack refer to the attack and nonattack comments, respectively.

We asked how pervasive the verbal attacks against overweight individuals are. Our data show that the majority of comments verbally attacked overweight people (56%, 174/316). Only 28% percent of the comments (89/316) defended the overweight individuals. Our analysis of RQ5b assesses to what extent verbal attacks toward overweight individuals are accompanied by swear words. When attacking (mean 0.37, SD 0.74), comment intensity (ie, number of swear words) is significantly higher than nonattack comments (mean 0.17, SD 0.40), *t*_314_=2.48, *P*=.01. RQ5c asks up to what comparative extent male and female overweight individuals receive comments that include swear words. A significant difference was found between comments that target overweight male individuals (mean 0.21, SD 0.50) and those targeting females (mean 0.38, SD 0.70), *t*_204.97_=2.040, *P*=.04 (Figure 1). Also, when targeting overweight females, attacking comments accompanied more swear words (mean 0.44, SD 0.77) than nonattack comments (mean 0.23, SD 0.48), t_204.97_=−2.040, *P*=.05. However, no difference between the attack and nonattack comments was found for those targeting males.

## Discussion

### Results and Explanations

The primary purpose of this study was to understand how and up to what extent overweight individuals are verbally attacked online. Particularly, this study delved into patterned differences in verbal attacks depending on the genders of the commenters and the overweight target, especially in terms of (1) the topic of the comment, (2) the intensity of derogation (ie, swearing), and (3) the targeted genders. These results are important for the development of intervention strategies that can effectively reduce the social stigma of obesity, which can adversely influence overweight individuals’ mental and physical health [4]. In our data, when addressing evolutionary mate values, both overweight men and women were verbally attacked for deficits in reproductive power, primarily signaled by physical attractiveness. This result is seemingly unexpected according to the evolutionary psychological view, which suggests that men will be attacked for deficits in men’s primary mate features: capacities for resource acquisitions (eg, financial prospect and industriousness). However, one may need to apply the evolutionary psychological view with caution. Buss (1989) argued that mate feature preferences for men and women vary depending on time, culture, and physical environment [21]. Historically men’s physical attractiveness may not have signaled economic or social status or ability to accumulate resources (eg, money) to raise offspring and protect a family. In contemporary society, however, men’s physical attractiveness may be an important visual cue. Physical attractiveness may signal an ability to get resources for supporting and protecting a family, as a good-looking man is more likely to be hired, make more sales, and receive raises [50]. Our results show this change in how men’s mate features are preferred. Another possible explanation for this finding may be that the comments were posted in response to videos about meeting a date through Tinder, a mobile app where men and women may look for a short-term relationship. In this instance, men’s resource acquisition may not be as highly valued since females may not be looking for a long-term relationship (in which resource acquisition would be more useful).

While there were no significant gender differences in the dichotomous mate values, our data do show gender discrepancies in the prominence of the topic of the verbal attacks. First, we explored up to what extent weight stigma is perpetuated within the comments on the 2 Tinder videos. Our data show that the majority of the comments attack overweight individuals rather than defend them. This result generally supports the view that *YouTube* is a space where people feel disinhibited to express hatred against others [38]. Given that the 2 Tinder videos do not explicitly ridicule or tease obese individuals, our result also confirms that even in cases of indirect address of obesity, commenters choose to verbally attack obese individuals. Some may argue that people, especially bored young people, enjoy verbally attacking targets with hostile language (ie, flaming) [38]. Some users may even think that flaming is a “funny way of interacting that is not to be taken too seriously or that it is a necessary side effect of vivid debate and freedom of speech” [38]. Importantly, people are likely unaware of what their targets―overweight individuals―feel about their aggressive comments, and the possible subsequent effects induced by such stigmatizing comments. This may imply that to stop anonymous aggressive comments, we need stricter self-regulation of websites. For example, Reddit, a popular social network website, has already started stricter regulation, closing hatred forums within its website including “Fat People Hate,” the biggest forum in Reddit at the time [51]. Of course, this restriction will likely prompt debates on freedom of expression. Nevertheless, our results, along with findings from other studies, elucidate a worrying situation in which the online world is filled with unfettered stigmatizing messages against overweight individuals.

Second, overweight men are more likely to be attacked for deficits in heterosocial skills than overweight women. For men, heterosocial skills may be seen as an important feature for success in mating [27], but overweight men are stereotyped as lacking in heterosocial skills just as other negative traits unjustly associated with overweight individuals. This stereotypical assumption may result in more verbal attacks targeting heterosocial skills of overweight men than that of overweight women. In addition, perhaps unexpectedly, overweight women were more likely to receive verbal attacks addressing capacities for resource acquisition (eg, being lazy, unhealthy) than men. This is also seemingly at odds with the evolutionary psychological perspective, in which men evolved to value female mates’ reproductive power. One possible explanation can be that contemporary overweight women are blamed more for failures in individual responsibility or ability to control or discipline her body than overweight men, that is, overweight women may be perceived to be less disciplined and weaker than overweight men. Some research supports this view suggesting that overweight women are seen to lack in ability to do a strenuous job, whereas overweight men are seen as capable as nonoverweight men [52]. However, this argument requires further validation based on more empirical research, including more representative samples of comments from *YouTube* or other digital platforms. Also, a survey with a representative sample of the population may be needed to examine changes in attitudes about evolutionary mate values for overweight men and women.

The third gender difference examined in this paper is the susceptibility to weight stigma. Is weight stigma, as many studies suggest, primarily a women’s issue even in a digital context? Our data suggests that overweight women are prevalently victimized by men. More specifically, men are more likely than women to attack overweight women, and women do not show any tendency of attacking overweight men more than women. It is possible that men may blame obese women for not conforming to their role of “being an attractive object” in a patriarchal society [32]. Also, we found that overweight women receive significantly more swear words within comments than overweight men. This is important as swearing can indicate anger, arousal, or intention to strengthen argument [53,54], and if being overweight for women is less “forgivable” than for men, then women would be attacked with higher levels of intensity, indicated by a higher number of swear words.

### Limitations, Implications, and Future Directions

This study has 3 limitations. First, our study is exploratory, focusing on only 2 sets of comments posted in response to the 2 Tinder date videos. Our findings cannot be generalized to all *YouTube* videos, which may differ in terms of their content (eg, narrative structure). Therefore, this study should be expanded into comments on other videos and social media platforms. Also, a larger dataset (ie, big data) from diverse sources can be used to further delve into the gender differences in verbal aggression against overweight individuals, increasing generalizability of the findings [55].

Second, this study sampled only root comments—comments that are posted in direct response to the video content and first response comments to the root comments. Therefore, our sampling methodology may exclude second, third, or later response comments that may provide further counterarguments against comments that attack overweight individuals. To understand how commenters may defend or empathize with overweight men and women, future research should include more response comments as well.

Third, researchers should seek to identify how overweight individuals respond to verbal attacks on *YouTube*. This research question would be difficult to answer through content analysis, as commenters rarely reveal their weight within this context. Survey or focus group interviews which ask questions about overweight individuals’ experience of verbal attacks on *YouTube* may be useful in exploring this question further. Also, our research focused on 2 binary genders (ie, male vs female). Thus, the impacts of nonconforming and marginalized gender identities of commenters or overweight targets (eg, transgender or nonbinary) await future research.

Despite these limitations, this study contributes to current research on weight-stigma by showing how weight-stigma is perpetuated *differently* for overweight men and women in a digital context. Theoretically, this study attempted to expand evolutionary psychological theories by examining gender differences within the context of weight stigma. Our results show that the evolutionary perspective on verbal aggression needs to be applied cautiously with overweight individuals in contemporary society.

Findings of this study should provide useful input in designing effective gender-tailored messages—customizing a message to different perceptions of overweight men and women—to maximize resources available to improve attitude toward obese individuals [56], and, in the long term, reduce depression caused by weight stigma in a digital context. For example, the message for overweight men should focus on the perception that overweight men lack in heterosocial skills by showing counterstereotypical images of overweight men (eg, overweight men being active and good at socializing with others). Conversely, messages for overweight women could better tackle the perception that overweight women lack in will power (eg, overweight women depicted as active in sports such as marathon that require greater will-power) rather than the negative traits primarily associated with overweight males. Also, based on findings of this study, we suggest that further research begin addressing gender discrepancies in weight stigma. For example, the widely used scale for weight bias, Fat Phobia Scale, was originally developed based on adjectives to describe overweight individuals, listed by people who entered a motor vehicle license bureau in a suburb of St. Paul/Minneapolis metropolitan area in 1984 [57]. This study identified 12 out of the total 14 items in this scale to be traits generally related to capacities for resource acquisition; only 2 items of this semantic differential scale (Item #3: “Unattractive vs Attractive”; Item#11: “Shapeless vs Shapely”) were related to reproductive power. Thus, this scale may need revision to incorporate a more appropriate balance of negative traits associated with reproductive power and capacities for resource acquisition. Additionally, more explicit items measuring overweight individuals’ heterosocial skills may need to be included as well. In other words, more gender-balanced weight bias scales are needed to reflect the changes in gender differences in weight stigma.

In conclusion, the results of this study were indispensable in that they revealed how implicit beliefs stigmatize overweight men and women differently. Thus, based on the data, it is recommended that effective intervention strategies that tackle these specific beliefs and gender be developed to lift the mental burden for overweight individuals in fighting obesity.

## Abbreviations

RQ: research question
